# Development of Antifouling Thin-Film Composite/Nanocomposite Membranes for Removal of Phosphate and Malachite Green Dye

**DOI:** 10.3390/membranes12080768

**Published:** 2022-08-07

**Authors:** Moucham Borpatra Gohain, Sachin Karki, Diksha Yadav, Archana Yadav, Neha R. Thakare, Swapnali Hazarika, Hyung Keun Lee, Pravin G. Ingole

**Affiliations:** 1 Chemical Engineering Group, Engineering Sciences and Technology Division, CSIR-North East Institute of Science and Technology, Jorhat 785006, Assam, India; 2 Academy of Scientific and Innovative Research (AcSIR), Ghaziabad 201002, Uttar Pradesh, India; 3 Biological Sciences and Technology Division, CSIR-North East Institute of Science and Technology, Jorhat 785006, Assam, India; 4 Technology Research Institute, QuantumCat Co., Ltd., Daejeon 34028, Korea

**Keywords:** polysulfone, UiO-66-NH_2_ nanoparticles, interfacial polymerization, malachite green, phosphate removal, antibacterial test, antifouling study

## Abstract

Nowadays polymer-based thin film nanocomposite (TFN) membrane technologies are showing key interest to improve the separation properties. TFN membranes are well known in diverse fields but developing highly improved TFN membranes for the removal of low concentration solutions is the main challenge for the researchers. Application of functional nanomaterials, incorporated in TFN membranes provides better performance as permeance and selectivity. The polymer membrane-based separation process plays an important role in the chemical industry for the isolation of products and recovery of different important types of reactants. Due to the reduction in investment, less operating costs and safety issues membrane methods are mainly used for the separation process. Membranes do good separation of dyes and ions, yet their separation efficiency is challenged when the impurity is in low concentration. Herewith, we have developed, UiO-66-NH_2_ incorporated TFN membranes through interfacial polymerization between piperazine (PIP) and trimesoyl chloride (TMC) for separating malachite green dye and phosphate from water in their low concentration. A comparative study between thin-film composite (TFC) and TFN has been carried out to comprehend the benefit of loading nanoparticles. To provide mechanical strength to the polyamide layer ultra-porous polysulfone support was made through phase inversion. As a result, outstanding separation values of malachite green (MG) 91.90 ± 3% rejection with 13.32 ± 0.6 Lm^−2^h^−1^ flux and phosphate 78.36 ± 3% rejection with 22.22 ± 1.1 Lm^−2^h^−1^ flux by TFN membrane were obtained. The antifouling tendency of the membranes was examined by using bovine serum albumin (BSA)-mixed feed and deionized water, the study showed a good ~84% antifouling tendency of TFN membrane with a small ~14% irreversible fouling. Membrane’s antibacterial test against *E. coli.* and *S. aureus.* also revealed that the TFN membrane possesses antibacterial activity as well. We believe that the present work is an approach to obtaining good results from the membranes under tricky conditions.

## 1. Introduction

Nowadays water scarcity has turned into a critical issue because of the increasing population and rising industrial growth. Hence, low-cost technologies are required which will be feasible for everyone. Although there are several technologies available in the market, but the low-cost and sustainable technology is membrane-based technology, especially based on nanofiltration (NF) membranes [1]. Nanofiltration membranes are pressure-driven membranes which are the intermediate of reverse osmosis membrane and ultrafiltration membrane. Nowadays, nanofiltration membranes are designed in many ways to have good permeability such as ultrafiltration membranes and good rejection such as reverse osmosis membranes [2]. The NF membrane technology is working in several areas such as wastewater treatment, seawater desalination, power generation, food processing, enantiomer separation etc. [3,4,5,6,7,8,9,10]. NF is also feasible because it is working on low pressure, so ultimately low energy consumption, low equipment cost, low fouling tendency, and high rejection for various components (contaminants) [11]. 

Dyes are one of the hazardous pollutants that pollute our water bodies of which the major source is industrial wastewater. Their toxin effect, carcinogenicity, visible colour, etc. damages the water bodies and makes them unpotable for daily purposes even in a small amount [12,13]. In the textile industries, the dye removal from the effluents is a major problem and they have to give too much importance to it. The presence of a high quantity of dyes in the water makes it more polluted and it makes adverse effects on the aquatic life by inhibiting their photosynthetic activity. Malachite green (MG, C_23_H_25_ClN_2_) is a synthetic dye commonly used in the food and cosmetic industries as a colouring agent. In the pharmaceutical industry also, MG is used as an antifungal and antibacterial agent [14]. MG is very toxic to aquatic and terrestrial animals and highly cytotoxic to mammalian cells and it is banned in several countries [15]. MG is not reduced because of its reduced form, leucomalachite green, which can cause mutation and act as a carcinogen. Due to these adverse effects to remove this dye the membrane base technology is a good choice. Recent studies on membranes for the removal of various dyes by using the nanomaterial incorporated membranes have been studied by researchers [16,17]. People used the adsorption for the removal of MG but required a large amount of materials for it. Considering the applications of the polymeric membrane, we used the molecular organic framework (MOF) incorporated TFN membrane to remove the MG. Thin-film nanofiltration membranes have attracted the promising attention of removing dye successfully in past decades [12,13,18,19]. 

Excess phosphates in wastewater can cause eutrophication of the water body, make the environment polluted and affect the body of a human. Phosphate removal from the wastewater especially from domestic wastewater is very important and it reduces the potential for eutrophication in receiving water. The adsorption of phosphate and dyes is one of the ways but nowadays removal of these compounds by using the polymeric membrane technology is the best way. For the large-scale, wastewater treatment plant to remove the phosphate and dyes required a low-cost, sustainable and continuous process and as per our findings, implementation of membranes-based technology is best for it [20]. Polymer membranes prepared with polysulfone polymer are the best choice for researchers as it is a high-performance thermoplastic having high transition temperature with the best mechanical strength and excellent thermal and chemical stability. Polysulfone can make the suitable base matrix by the phase inversion process [21,22,23]. Nanoparticles incorporated in thin-film nanocomposite membranes are a good choice for the separation of contaminants from the water. There are several membranes have been prepared by the researchers to remove metal ions such as Ni (II), Co (II), Cd (II), Cu (II), Cr (VI), Pb (II) including phosphate and nitrate from aqueous systems [24]. However, achieving good separation efficiency at a low concentration level is a challenging task. Compared with the thin-film composite membrane TFN membranes give improved performance in the form of permeability, selectivity and antifouling properties [25].

There are several conventional as well as modern ways of incorporating nanomaterial to form nanofiltration membranes. Interfacial polymerization by aqueous and organic phase coating is an assuring method to fabricate thin-film membranes [26]. In a work by Sutedja et al., they prepared a TFC membrane by interfacial polymerization on the PSf support for separating the textile dyes from wastewater and achieved 88% dye rejection [27]. An improvement in the separation abilities of the membranes can also be attributed to modifying the polysulfone support. Peyravi et al., showed adding sulfonated poly(ether sulfide sulfone) (SPESS) copolymer into polysulfone polymer through blending has improved the performance of prepared TFC-SRNF (solvent resistant thin-film composite nanofiltration) membrane [28].

Fouling is a major drawback of membrane separation processes; it affects the longevity and reduces the performance of the membrane. Big molecular weight proteins slowly adsorb on the membrane surface with time and create a layer which diminishes the membrane activity. A lot of focus has been put by the researchers on mitigating the fouling of membranes. Hydrophilic modification of the membrane increases the hydrophilicity of the membrane and shows less affinity towards the organic molecules and foul less. Blending and surface modification are two ways to impart hydrophilicity. Surface modification leads to the formation of a hydrophilic layer on the surface [29]. Zhang et al. in their work grafted a zwitterion poly(sulfobetaine methacrylate) (pSBMA) on the surface of a polyamide membrane via surface-initiated atom transfer radical polymerization. The prepared modified membrane exhibited ~97% reduction of protein adsorption and ~65% increased water permeability [30]. Researchers have also used the interfacial polymerization method to generate a polyamide layer which increases the hydrophilicity. Mixing nanomaterial also increases the hydrophilic nature of the mixed matrix as well as thin-film nanocomposite membranes. Nanomaterials such as graphene oxide (GO) nanoplates, cellulose nanocrystals, and kaolin are well-known materials used for synthesizing TFN membranes. Such membranes showed good hydrophilicity and were used for the separation of metal ions from the water [31,32,33]. Metal-organic frameworks (MOFs) are one of the promising classes of porous crystalline materials its use in the TFN membranes has to make high permeance and greater rejection membrane [34]. UiO-66-NH_2_ is a kind of molecular organic framework (MOF) which have an average size of ~100 nm and showed good enhancement in the separation ability of the membranes. researchers have used this material for brackish water, and seawater desalination [35].

In this work, we have prepared the TFC and TFN membranes for separating malachite green dye and for the challenge of removing phosphate ions from a low-concentrated aqueous solution. Owing to the above advantages of polysulfone (PSf), an attempt has been made to modify the internal microstructure and surface morphology of PSf membranes by introducing UiO-66-NH_2_ with piperazine (PIP) and trimesoyl chloride (TMC). The developed TFC and TFN membranes have been characterized by techniques such as Scanning electron microscopy (SEM), Fourier Transform Infra-Red (FTIR) spectroscopy, Thermogravimetric Analysis (TGA), Contact Angle (CA), and X-ray Photoelectron Spectrometer (XPS) techniques.

## 2. Experimental 

### 2.1. Materials

For the preparation of the membranes polysulfone polymer (Mw ~ 35,000) was purchased from M/s Sigma Aldrich Chemical Company, St. Louis, MO, USA. The thin-film preparation was carried out by using piperazine (purity = 99%), trimesoylchloride (TMC) (purity = 98%) purchased from M/s Sigma Aldrich and n-hexane (purity = 99.9%) used for dissolving TMC is purchased from M/s RANKEM range of laboratory chemicals, Gujarat, India. For preparing the nanomaterials Zirconium (IV) chloride (ZrCl_4_) (purity ≥ 99.9%) and 2-aminoterephthalic acid (purity = 99%) was also purchased from M/s Sigma Aldrich. The dimethylformamide (DMF) (purity = 99%) was purchased from Finar Limited, Gujarat, India for use in nanomaterial preparation and as a solvent of polymer dope solution. For preparing the feed solution the malachite green dye was purchased from GLR innovations and for the phosphate feed solution potassium dihydrogen phosphate (KH_2_PO_4_) (purity = 99.5%) was bought from SRL Pvt. Ltd. Mumbai, Maharashtra, India. For adjusting the pH of the phosphate feed solution, the 30–40% concentrated hydrochloric acid (HCl) was purchased from M/s RANKEM. For the antifouling study the BSA (Purity: 96–98%) of ~66 kDa, CuSO_4_ (Purity: ≥99%), Na_2_SO_4_ salt (Purity: >99.5%) were purchased from SRL Pvt. Ltd., MERCK group chemical companies, Bengaluru, India, and TCI Co., Ltd., Chennai, India, respectively.

### 2.2. UiO-66-NH_2_ Nanomaterial Preparations

The UiO-66-NH_2_ nanoparticles are prepared by the already reported method [36,37]. ZrCl_4_ (1.05 g) and 2-aminoterephthalic acid (NH_2_-H_2_BDC) (1.56 g) were dissolved in 40 mL of dimethylformamide (DMF) by stirring and sonication. After 30 min 17 mL acetic acid was added to the solution and dissolved again for 15 min. The solution was then transferred into the autoclave for a solvothermal reaction at 140 °C for 24 h. After that, the resulting material was washed by DMF, and the obtained powder was then set to dry for 7–8 h. The obtained nanomaterial was tested by FE-SEM, FTIR and XRD analysis for its characterization.

### 2.3. PSf Membrane Preparation

The PSf membranes were prepared by using the most common non-solvent induced phase separation (NIPS) method. An 18% solution of polysulfone polymer was prepared in N,N-dimethylformamide (DMF) solvent after stirring at 70 °C for 6–7 h. The dope solution is then set to cool down at normal temperature for removing the moisture or bubble present on it. The dope solution was cast on polyester fabric support using a casting knife and immersed immediately into the non-solvent water bath. The process of phase separation dissolves the DMF into the water and caused the solidification of a polymer leading to the flat sheet polysulfone (PSf) membranes. The membrane is washed and placed into the deionized water until the next use.

### 2.4. Preparation of M1 and M2 Membrane

**Figure 1:**
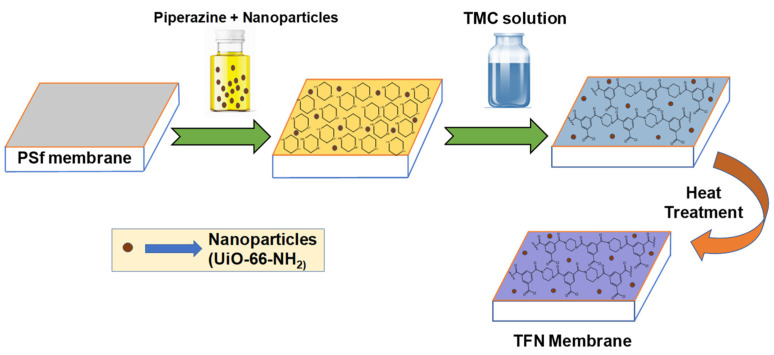
Representation of preparation of TFN (thin-film nanocomposite) layer on the surface of PSf membrane (Polysulfone-membrane) via interfacial polymerization method.

Two different kinds of membranes were prepared by using interfacial polymerization on the polysulfone membrane surface. Simple TFC and TFN membranes were synthesized for the comparative study. For modifying the surface, 2 wt% piperazine (PIP) aqueous solution and 0.2 wt% TMC in n-hexane solution were prepared. For the development of the TFN membrane, UiO-66-NH_2_ (0.02 wt%) nanoparticles were mixed in the aqueous phase monomer. To prepare the thin selective layer the PSf membranes were first coated with an aqueous phase by immersing in the solution for 10 min. After that, the membranes were drained off for a time to remove the additional water on the membrane surface. The PIP coated membrane was then immersed in the TMC containing n-hexane organic solution where the interfacial polymerization took place for 1 min and a thin film was constructed on the PSf membrane’s surface. The TFC and TFN membranes are then subjected inside the oven for 10 min at 65–70 °C. A schematic representation of preparing the TFN membranes is shown in Figure 1. The TFC and TFN membrane’s preparation and composition are summarized in Table 1. 

### 2.5. Material and Membrane Characterization Techniques

The Attenuated Total Reflectance Fourier Transform Infrared (ATR-FTIR) is an analytical technique that is used to detect organic, inorganic, and polymeric materials. Infrared light is used in this method to scan test samples and observe chemical properties. Using Perkin Elmer, System 2000 Infrared spectrophotometer, Massachusetts, United States America, wave number measured as frequency over the range 4000–400 cm**^−^**^1^. Analysis for XPS-using Thermo-Fisher-Scientific: ESCALAB Xi MA, USA, was carried out and is used for measuring the composition, pragmatic formula, and electronic and chemical state of the elements inside the materials. The morphology and internal structure of the composite membranes were examined by FE-SEM. FE-SEM analysis results to show surface anatomy and their cross-section used the instrument Carl ZEISS Microscopy, Germany. For the calculation of phosphate (the molybdenum blue method) and malachite green dye rejection, the absorbance amount was determined by UV-Visible spectrophotometer, Specord 200 of Analytik Jena, Jena, Germany. The contact angle analysis was carried out using KYOWA (Interface Science Co. Ltd. Tokyo, Japan), Model-DM-501, to measure hydrophilic and hydrophobic characteristics of the membrane by determining angle. TGA was performed, and heated the samples to 700 °C, at a heating rate of 10 °C min**^−^**^1^, in an N_2_ atmosphere. TGA data were used to determine the weight of the encapsulated extractant per unit weight of PSf.

### 2.6. Permeability and Removal Test

**Figure 2:**
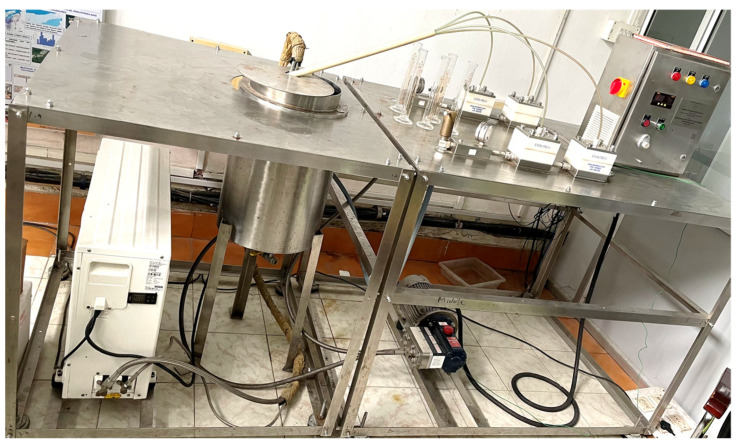
The crossflow NF/RO (Nanofiltration/Reverse Osmosis) four-cell membrane testing unit.

The performance of the synthesized M1 and M2 membranes was tested in the crossflow NF/RO four-cell membrane testing unit provided by M/s. Prova Pvt. Ltd. Mumbai, India, shown in Figure 2. The membranes were subjected to the crossflow cells and first run with deionized water for 2 h to mitigate the experimental errors. Two different feed solutions; a 100-ppm aqueous solution of malachite green dye and a 10-ppm aqueous solution of KH_2_PO_4_ (phosphate feed) were prepared. The pH of the KH_2_PO_4_ feed solution was decreased to 4 from 7 by adding 0.1 N concentrated HCl dropwise. Membranes are tested for 8–10 h at two different pressure ranges; 10 bar and 15 bar. The permeability was calculated by measuring the permeate volume each hour using Equation (1). UV-spectrophotometer is used for measuring the occurred rejection in the permeate for malachite green and Equation (2) is used for calculating percentage rejection.

(1) $$\mathrm{Permeation}=\frac{\mathrm{Volume}}{\mathrm{Area}\;\times \mathrm{time}}$$

The unit of the flux is Lm^−2^h^−1^.

(2) $$\%\;\mathrm{Rejection}=\frac{A_{f}-A_{p}}{A_{f}}\times 100$$

where A_f_ is feed-absorbance and A_p_ is permeate-absorbance, measured at ~617 nm. 

The adsorption study for 10 ppm phosphate solution (by dissolving KH_2_PO_4_ in deionized water) has been carried out. The molybdenum blue method [38] is used to measure the adsorption/rejection efficiencies via UV-visible spectrophotometry.

### 2.7. Antifouling Testing of Membrane

The antifouling capabilities of prepared membranes were examined by testing them against a mixture of 500 ppm BSA + 25 ppm Na_2_SO_4_ + 25 ppm CuSO_4_ aqueous solution (Mix feed) and deionized water (DI) at 10 bar pressure. The permeation of the membranes was first examined every 30 min with DI water for 90 min. The obtained average pure water permeation value was noted as J_w,1_. Then the Mix feed was filled in the feed chamber and membranes were tested for 90 min. The permeation value was measured at every 30 min and their average was noted as J_m_. After that DI water permeation was measured again which was noted as J_w,2_. This was run for 450 min and permeation values were measured. Using Equations (3) and (4), the total fouling percentage (F_T_) and flux recovery ratio (F_RR_) were calculated. After 360 min the membranes were washed with deionized water for 1 h to remove the fouling and then the DI water permeation was measured again, the final water flux was noted as J_w,f_. The permeation value against Mix feed before washing was considered final mixed feed flux J_m,f_. With the help of Equations (5) and (6) the reversible (F_R_) and irreversible fouling (F_IR_) that occurred on the membrane during complete testing was calculated.

(3) $$\mathrm{Total}\;\mathrm{Fouling}\;\left(F_{T}\right)=\frac{J_{w,1}-J_{m}}{J_{w,1}}\times 100$$

(4) $$\mathrm{Fouling}\;\mathrm{recovery}\;\mathrm{ratio}\;\left(F_{\mathrm{RR}}\right)=\frac{J_{w,2}}{J_{w,1}}\times 100$$

(5) $$\mathrm{Reversible}\;\mathrm{Fouling}\;\left(F_{R}\right)=\frac{J_{w,f}-J_{m,f}}{J_{w,1}}\times 100\;$$

(6) $$\mathrm{Irreversible}\;\mathrm{Fouling}\;\left(F_{\mathrm{IR}}\right)=\frac{J_{w,1}-J_{w,f}}{J_{w,1}}\times 100$$

### 2.8. Antibacterial Activity Testing

The antibacterial activity testing of the prepared membranes is carried out by the plate colony-forming count method. Nutrient agar aqueous homogeneous solution was prepared and set into the autoclave to sterilize. Then after the agar solution was poured into the glass petri dish which later becomes solidified, similar to gel. Nutrient agar is the food for the bacteria colony growth. The M1 and M2 membrane pieces were first sterilized by cleaning with 70% ethanol solution and exposing them to UV light. Then they are set on the agar Petri dishes and cultured Gram-positive bacteria *S. aureus* (*Staphylococcus aureus*) and Gram-negative bacteria *E. coli.* (*Escherichia coli*) was spread on the membrane’s surfaces. At one part of the petri dish aminoglycoside antibiotic named “Neomycin” was dropped a little for reference. The petri dish was then set to overnight incubation at 37 °C with a rotary shaker.

## 3. Results

### 3.1. Characterization

#### 3.1.1. Nanomaterial Characterization

**Figure 3:**
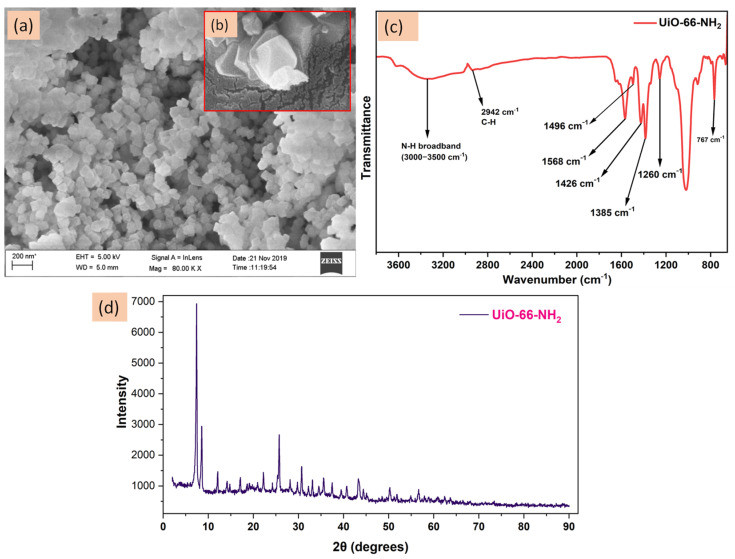
The obtained (**a**) FE-SEM image at 200 nm scale, (**b**) FE-SEM image showing the shape of UiO-66-NH_2_, (**c**) ATR-FTIR spectrum for UiO-66-NH_2_ nanoparticles and (**d**) P-XRD pattern obtained for UiO-66-NH_2_.

The synthesized nanomaterial UiO-66-NH_2_ was characterized using the ATR-FTIR and FE-SEM to have an idea about its size, shape and functionality on it. Figure 3a represents the FE-SEM image of the prepared nanomaterials at 80 K magnification.

It is clear from the image that the prepared nanomaterials are very fine and have sizes ~100 nm. The UiO-66-NH_2_ nanoparticles are octahedral in shape which can be visualized in their FE-SEM image in Figure 3b [39]. Figure 3c is the ATR-FTIR analysis. Different characteristic peaks are obtained which are mostly due to the organic part of the nanomaterial. The Zr-O peak which was obtained around 490 cm^−1^ was not depicted in the spectrum. Strong broadband from 3000–3500 cm^−1^ was observed due to the N-H stretching and its bending falls under 1500–1600 cm^−1^. The peaks around 1260 and 1384 cm^−1^ can be attributed to C-N stretching between aromatic carbon and the NH_2_ group. The C-N bending peak at 767 cm^−1^ is also present which is due to the C-N bending. The characteristic C-H stretching peak is obtained at 2942 cm^−1^. The COO^− ^ group present in the nanomaterial due to the added 2-aminoterephthalic acid shows characteristic peaks of asymmetrical stretching at 1496 and 1568 cm^−1^ while the symmetrical stretching peaks are obtained at 1426 and 1385 cm^−1^ [40,41]. Figure 3d is the obtained pattern of the powder-XRD analysis of UiO-66-NH_2_ nanoparticles. The obtained sharp lines in the pattern exhibit their high crystalline nature [42]. The FE-SEM, FTIR and XRD patterns provide the confirmational idea about the nanomaterial. As reported by Aghajanzadeh et al. in their research article UiO-66-NH2 nanomaterial provides a surface area of ~1258 m^2^/g and a pore volume of ~0.51 cm^3^/g exhibits that UiO-66-NH_2_ nanomaterials have a high surface area which is very beneficial for the membrane’s hydrophilic surface modification [43].

#### 3.1.2. Membrane Characterization

**Figure 4:**
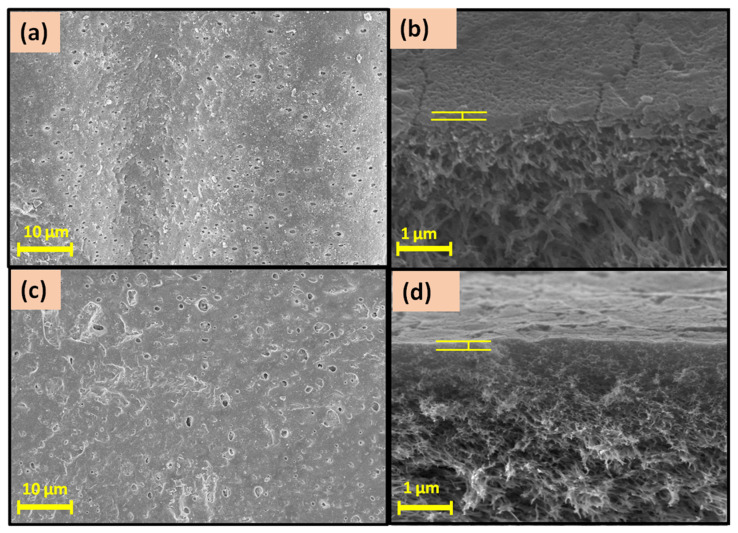
FE-SEM images of (**a**) surface view of M1 and (**c**) surface view of M2 membrane and the cross-section images of (**b**) M1 membrane and (**d**) M2 membrane.

The prepared TFC and TFN membranes were characterized by several spectroscopic and non-spectroscopic techniques to analyze their composition, construction, and surface and have an idea about their performance behaviour. The obtained FE-SEM images of the prepared TFC and TFN membranes are shown in Figure 4. On comparing the TFC (M1) and TFN (M2) membranes FE-SEM images, one can see better structures and more layer formation on the M2 membrane also a greater number of pores have been covered and reduced to a shorter diameter by the layer in M2 membrane. The (b) and (d) images show the cross-section of the prepared M1 and M2 membrane, respectively. 

Most of the cross-section image part was covered by the PSf and the porous inside structure is visible through the image. The porous structure allows the water molecules to pass inside the membrane and the surface-active thin-film hydrophilicity determines the water permeation behaviour. The more hydrophilic surface layer will have higher permeation which was measured by the water contact angle with the surface. By using the sessile drop method, the water contact angle of the prepared membrane’s surface was analysed. Table 2 represents the contact angle values and corresponding images of the prepared membrane. For comparing and understanding the effect of thin-film formation and nanomaterial incorporation the contact angle of the pristine PSf membrane was also measured. The polyamide layer was constructed on the PSf membrane surface in the M1 membrane which imparts polarity due to the amide group present on it and enhances the interaction with the water. It can also be seen with the dropped contact angle value of the TFC membrane compared to PSf membrane. The nanomaterials are functionalized with -NH_2_ groups which are known for creating hydrogen bonding with the water molecules thus their addition further enhances the surface hydrophilicity. As seen for the M2 membrane the water contact angle has dropped lower than the M1 membrane showing the effect of incorporated nanomaterials. The nanomaterials have also consisted of aromatic rings which are water repellent due to their hydrophobic behaviour and the concentration of nanomaterial was very small due to which a greater drop in the contact angle was not observed [44].

**Figure 5:**
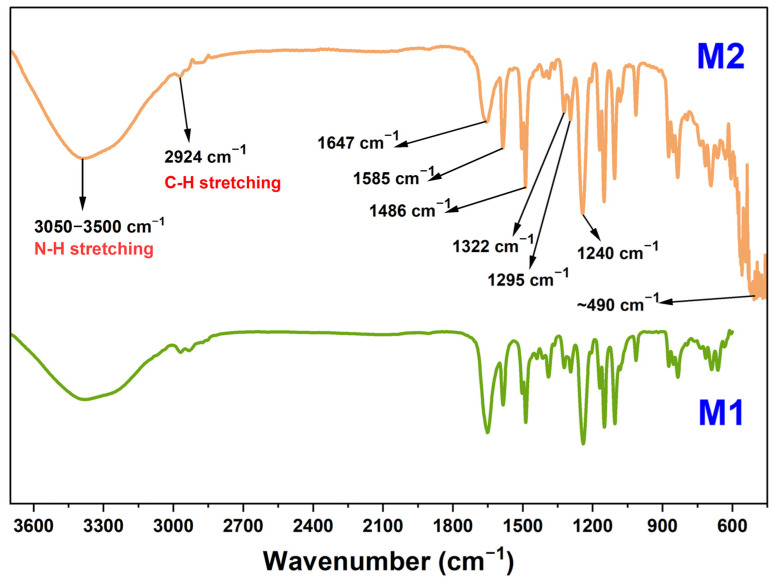
ATR-FTIR spectra of prepared M1 and M2 membrane.

The greater hydrophilicity on the surface is determined by the polar groups on the surface of the prepared membrane. Functional groups and other polar bonds with a certain dipole moment vibrates when subjected to IR light with different frequency. FTIR analysis technique is utilized to read and understand these vibrational motions and depict the type of bond. Figure 5 represents the ATR-FTIR spectra of the prepared M1 and M2 membranes. Most of the peaks obtained for the M1 and M2 membranes are common as both have a thin film layer made of polyamide and the same polysulfone support. Nanomaterials are the added extra materials in the M2 membranes and the characteristic peak of Zr-O due to those UiO-66-NH_2_ particles is visible ~490 cm^−1^ in the M2 membrane [45]. The carbonyl peaks at 1647 cm^−1^ and the broadband of N-H stretching at 3050–3500 cm^−1^ confirms the presence of the thin polyamide film on both the membranes. The peaks at 1486 cm^−1^ and 1585 cm^−1^ occurred due to the aromatic C-C stretching which is present in the thin film as well as in the nanomaterials and polysulfone support. The strong peak at 1240 cm^−1^ is attributed to the C-O-C bond and the peaks at 1295 cm^−1^, and 1322 cm^−1^ are denoted by asymmetric stretching of the O=S=O group. The peak around 2924 cm^−1^ is the significant peak of C-H stretching [46].

**Figure 6:**
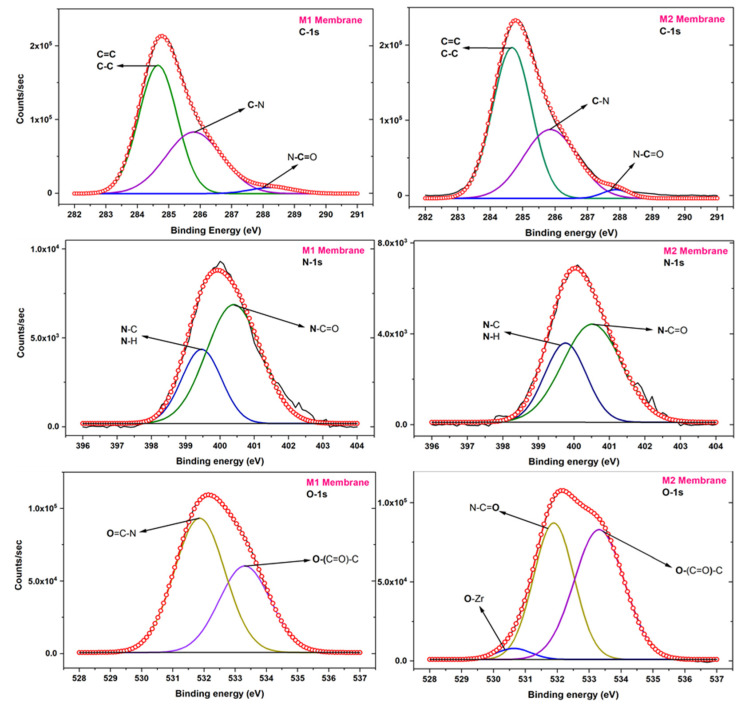
The high-resolution XPS plots of carbon, nitrogen and oxygen elements for M1 and M2 membranes.

X-ray photoelectron spectroscopy (XPS) analytical tool is used for elemental compositions. It also gives insight into the types of bonds on the surface. The prepared membranes are examined by XPS. The different high-resolution XPS graphs of carbon, nitrogen and oxygen elements are deconvoluted for the M1 and M2 membrane and represented in Figure 6. Different peaks of an element arise due to different surroundings present in the thin film. In the C-1s deconvoluted graph three major peaks around ~284.6 eV, ~285.9 eV and ~287.8 eV are fitted for carbons of C=C/C-C, C-N and N-C=O of amide, respectively. The polyamide thin-film possesses the nitrogen element and peaks due to the nitrogen of N-C/N-H and N-C=O bonds are shown at ~399.6 eV and ~400.4 eV for both the membranes [47]. 

Both the membranes differ in the nanomaterials which contain the Zr-O bonds characteristic of the UiO-66-NH_2_ nanoparticles. While assigning the peaks on the O-1s deconvoluted XPS spectrum an extra peak for the O-1s of the O-Zr bond has been represented in the M2 membrane [48]. The peak of O-1s of the O-Zr bond is at ~530.5 eV with low intensity as the nanoparticle’s concentration is small. Other O-1s peaks due to O=C-N and O-(C=O)-C are denoted at ~531.9 eV and ~533.6 eV. The represented O=C-N bond formed during the interfacial polymerization while the left acyl chloride group which has found a water molecule before the amine monomer has a reaction and hydrolysed and formed acid group shown by O-(C=O)-C. The UiO-66-NH_2_ contains many oxygens associated with the O-(C=O)-C group as well which also contributes in the case of the M2 membrane. 

**Figure 7:**
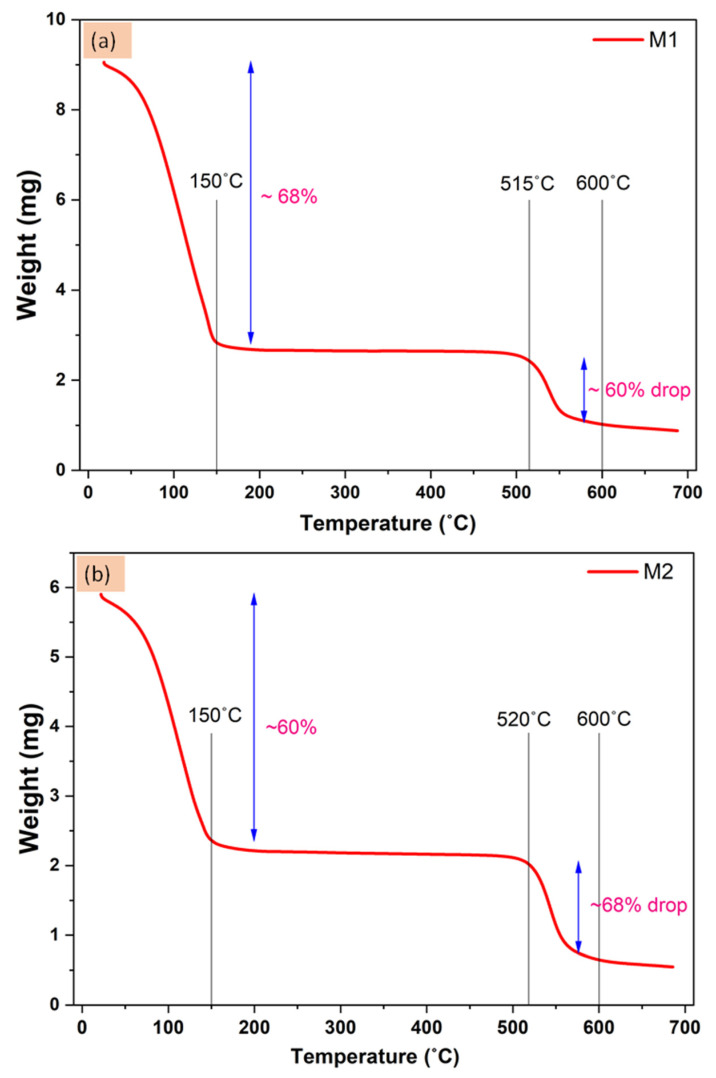
TGA analysis graph of the prepared (**a**) M1 and (**b**) M2 membrane.

The thermal stability of the prepared membranes was also tested up to 700 °C. The TGA analysis report of the prepared membranes is shown in Figure 7. Both the M1 and M2 membranes have shown an initial weight loss of 150 °C which is mostly due to the volatile matter and water moisture. Almost 60–68% of the weight was removed until 150 °C which may be due to the polymeric membranes may have gotten wet during the testing. After that, both the membranes are well stable up to 515–520 °C. The decomposition of the polymeric material and organic molecules begins after it and up to 600 °C, the M1 membrane showed a ~60% weight drop (from 515 °C to 600 °C) and the M2 membrane showed a ~68% weight drop (from 520 °C to 600 °C). The M2 membrane has a greater drop compared to the M1 membrane which is due to the added nanomaterials. The UiO-66-NH_2_ nanoparticles also have the organic aromatic part which also decomposes between 500–600 °C, because of this there is observed a higher % drop in the weight of the M2 membrane [49].

The observed graphs have displayed the thermal report of the prepared membranes as shown in Figure 7, however not a significant difference has been observed between M1 and M2 membranes. One can predict the M2 membrane is more thermally stable as it decomposes at 520 °C which is 5 °C greater than the M1 but such a small difference is not very significant. Most of the parts of the M1 and M2 membranes are the same and the added nanomaterials are very less in numbers as well. 

**Figure 8:**
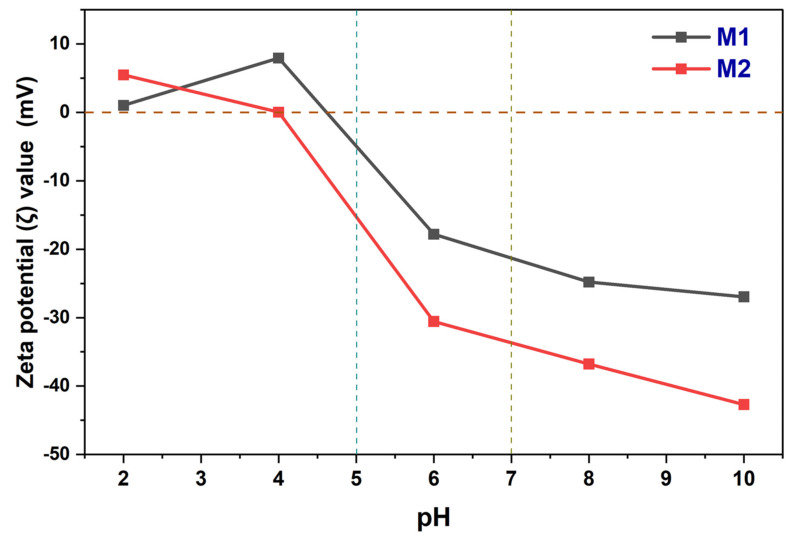
Zeta potential (ζ) observations of M1 and M2 membranes.

In the case of nanofiltration membranes, while performing ion separation, the surface charge value of the membranes affects their performance as the Donnan ion effect comes into prominent action. The surface charge value was measured with the help of zeta potential (ζ) and the prepared membranes M1 and M2 were analysed for it. The obtained graph from the zeta potential values of the membranes is shown in Figure 8. The figure depicts both the membranes having isoelectric points between pH 4 and pH 5. Above this point, the membranes are exhibiting a negative zeta potential value showing their anionic behaviour. The M2 membrane showed a higher negative zeta potential (ζ) value which can be considered due to the added nanomaterial (UiO-66-NH_2_). The polyamide layer built on the surface of both the membranes exhibits anionic behaviour due to the amide functional group, on the above, the added UiO-66-NH_2_ nanomaterial is rich in oxygen content which provides extra negative charge to the M2 membrane over M1. A higher charged surface gives rise to a stronger Donnan ion effect on the membrane. Considering the older membranes prepared using UiO-66-NH_2_ and COOH-TiO_2_, the currently prepared membranes showed excellent negative charge potential [1,50]. 

## 4. Discussion

### 4.1. Removal of Malachite Green Dye by TFC and TFN Membranes 

**Figure 9:**
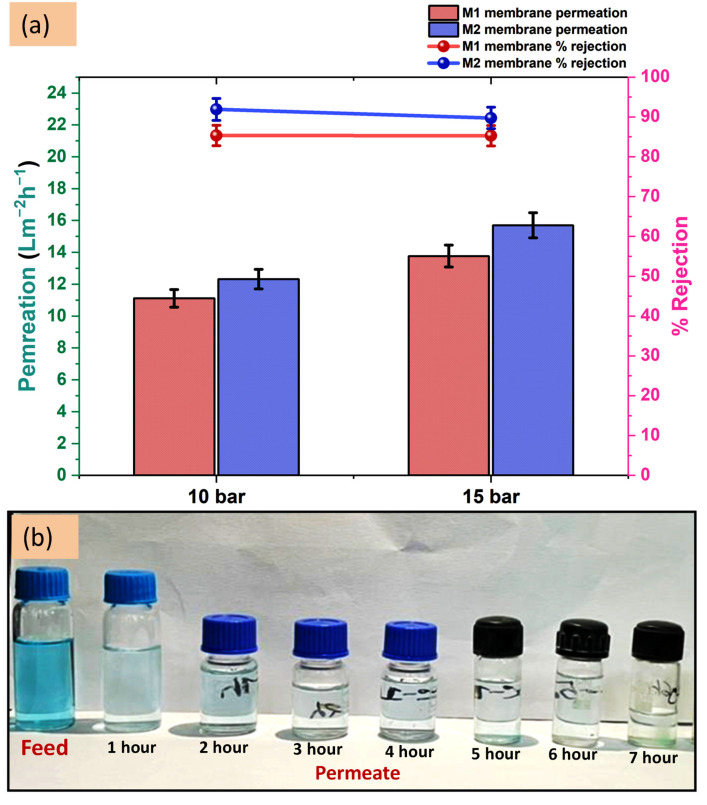
(**a**) M1 and M2 membrane’s permeation and rejection graphs against malachite green dye. (**b**) The images show the comparison of feed and obtained permeate per hour up to 7 h.

The prepared TFC and TFN membrane’s performance in removing 100 ppm malachite green is evaluated by testing in four cell membrane testing units. The obtained results are represented by a graph in Figure 9a and images are shown in Figure 9b. The membranes are tested at two different pressure 10 and 15 bar to understand the effect of pressure on the membrane performance. At 10 bar pressure the membranes M1 and M2 have shown 11.11 ± 0.5 and 13.32 ± 0.6 Lm^−2^h^−1^ permeability, respectively. The M2 membrane has higher permeability which is due to its more hydrophilic surface as explained by the contact angle experiment. The percentage rejection of malachite green by the M2 membrane is 91.90 ± 3%, which is higher than the M1 membrane having 85.34 ± 3% rejection. The M2 membrane differs from the M1 membrane only by the added nanomaterials and thus the difference in the percentage rejection value can be attributed to them. However, the difference is not large, yet it has increased the rejection by >90%. For a dye similar to malachite green which is a big size molecule, the separation mainly occurs by the size exclusion phenomenon. In addition, we have seen in the FE-SEM images the pore sizes of the M2 membranes are covered and reduced to smaller sizes due to the added nanomaterials. The nanomaterials are added to enhance the performance of the membranes and the obtained results are proving their effect even at higher pressure. When the pressure was raised to 15 bar the permeance increased as expected, but the percentage rejection decreased by a small margin. This is caused by the increased pressure which increases the pore size of the membrane that allows more permeation of the feed solution yet the rejection ability which is dependent on size exclusion and Donnan ion effect decreases. The results at 15 bar exhibit 13.77 ± 0.7 and 15.70 ± 0.8 Lm^−2^h^−1^ permeability from M1 and M2 membrane, respectively with corresponding 85.30 ± 3% and 89.74 ± 3% dye rejection. We observed negligible decrement in the percentage rejection of the M1 membrane when pressure increased to 15 bar but ~2% decrement occurred for the M2 membrane and the permeability of both the membranes have increased ~23–28%.

As for the high molecular weight dyes and proteins, size exclusion-based separation remains prominent. This is an assuring point for the prepared membranes that they can better remove the higher molecular weight dyes than malachite green. For example, Li et al. reported the preparation of UiO-66-NH_2_/GO composite nanofiltration thin-film membrane, which they used for removing cationic methylene blue (MB), neutral rhodamine B (RB) and anionic Congo red (CR) dyes [51].

### 4.2. Removal of Phosphate by TFC and TFN Membranes

**Figure 10:**
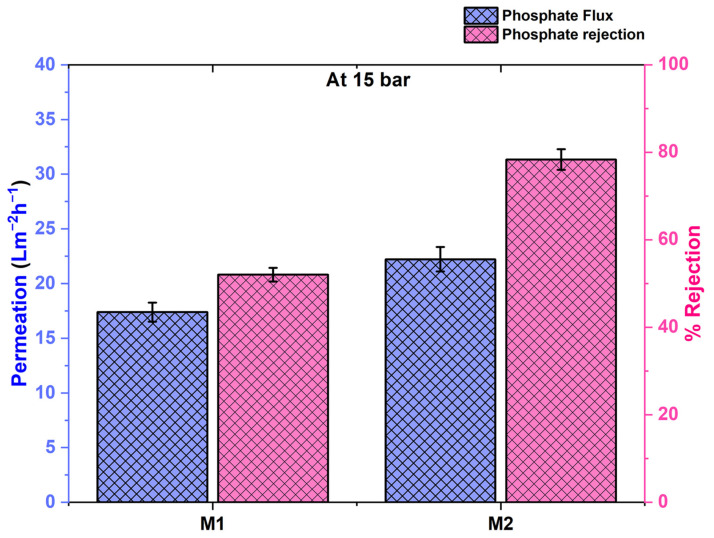
Permeability and rejection performance of M1 and M2 membrane at 15 bar pressure against phosphate.

The phosphate removal ability of the membrane has also been tested at 15 bar pressure. The plotted graphs for the M1 and M2 membrane’s permeation and rejection have been represented in Figure 10 with cross-pattern bar diagrams. The M1 membrane has shown 17.39 ± 0.9 Lm^−2^h^−1^ permeability with 52.05 ± 3% rejection of the phosphate ions. Meanwhile, the nanomaterial-loaded M2 membrane showed 22.22 ± 1.1 Lm^−2^h^−1^ permeability with 78.36 ± 3% rejection. The permeability of the M2 membrane is greater than the M1 due to the greater surface hydrophilicity and it also exhibits the greater rejection of phosphate ions. The difference between the percentage rejection is significantly bigger as the M2 membrane has exhibited ~26% greater separation. The Donnan ion effect works effectively against ions and the added nanomaterials contain oxygens that make the membrane surface more negative which was already been negative due to the polyamide layer at neutral pH. Adding the UiO-66-NH_2_ nanoparticles which have metal oxide bonds (Zr-O) and are polar in nature have enhanced the Donnan phenomenon. Due to this, the M2 membrane’s surface is more negatively charged as seen in the zeta potential graph in Figure 8 and the rejection of phosphate ions has occurred more.

The obtained results of phosphate removal seem weak if we look at the rejection of the dyes carried out by the same membranes. One thing also to be noticed here is that the feed concentration was only 10 ppm while phosphate separation, which is very small compared to the 100 ppm feed solution while separating the dye. Removing the impurity from a less concentrated solution is always harder and also the different phenomenon (Donnan ion effect) is prominent for phosphate separation.

### 4.3. Antifouling Study of the Membranes

**Figure 11:**
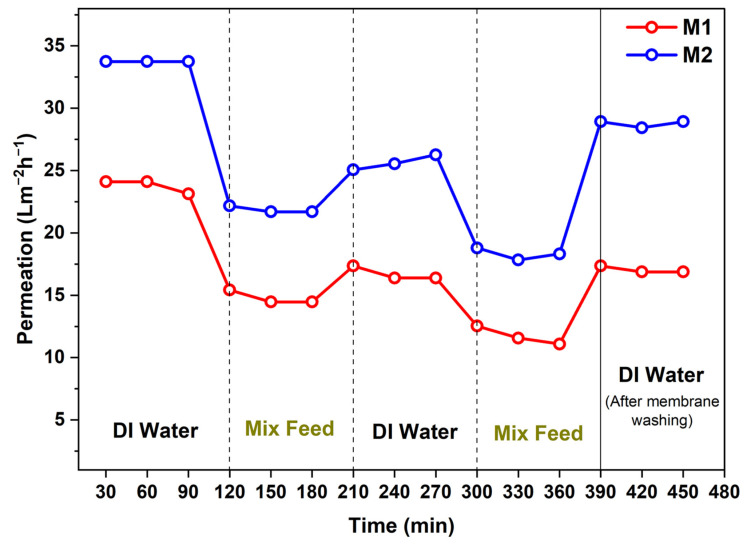
Effect on the permeation of membranes after exposure to fouling materials (in mix feed) for 480 min.

Antifouling studies have been carried out to test the tendency of membrane surfaces to stay clean from fouling. Organic proteins are mostly the molecules that cause the fouling therefore for the testing BSA protein is used along with Na^+^ and Cu^2+^ ions. The variation in the permeation value with time has been represented in Figure 11. As can be seen in Figure 11 the permeation value decreased against mix feed and again increases when conducted with deionized water (DI). Membranes are alternatively treated with deionized water and mixed feed solution to check the total fouling (F_T_) and flux recovery ratio (F_RR_) that occurred in a particular cycle.

**Figure 12:**
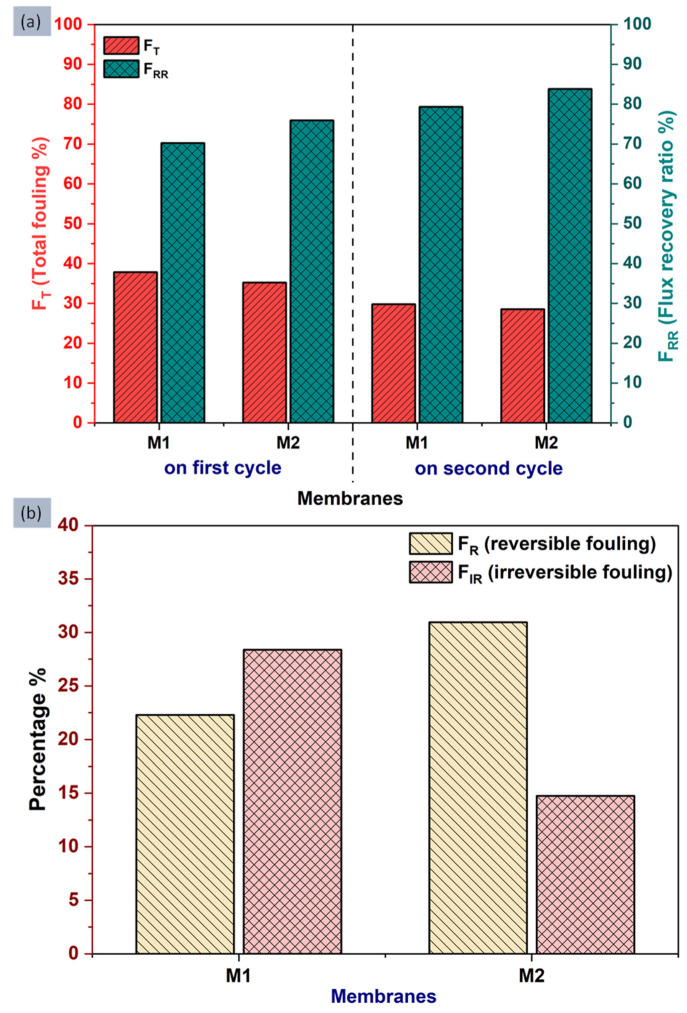
Different fouling parameters were obtained for the prepared membranes (M1 and M2) showing (**a**) Total fouling (F_T_), Flux recovery ratio (F_RR_) and (**b**) reversible fouling (F_R_) and irreversible fouling (F_IR_).

Figure 12a shows the bar graph of total fouling (F_T_) and flux recovery ratio (F_RR_) observed for the M1 and M2 membranes in two consecutive cycles. F_T_ is the measurement of the total fouling that occurred in the membranes and F_RR_ is the measurement of total recovered water flux after fouling, it is also referred to as the “antifouling tendency” of the membrane. During the first cycle, the TFC M1 membrane exhibited 37.8% total fouling with 70.2% flux recovery, while the TFN M2 membrane showed a smaller F_T_ value of 35.2% only, and a greater F_RR_ value of 75.9%. In the second cycle, the F_T_ value has been decreased by 7–9% and the F_RR_ value increased by 6–10% for both M1 and M2 membranes still the M2 membrane has a low total fouling percentage (FT) of 28.5% and a high flux recovery ratio or antifouling tendency (F_RR_) 83.8%. The decrement in the total fouling and flux recovery ratio can be attributed to the added nanomaterials. Even the contact angle values have demonstrated that the M2 membrane has a greater hydrophilic nature which also confirms the point that more hydrophilic membranes are less prone to organic fouling [1].

The total fouling of the membrane can be considered of two types one is reversible fouling (F_R_) and another is irreversible fouling (F_IR_). Reversible fouling (F_R_) was easily washed away with normal water cleaning and for that, the membranes were dipped into the water after 360 min, for 1 h to remove the fouling. F_R_ and F_IR_ are the measurements which represent reversible fouling and irreversible fouling, respectively. The obtained and calculated percentage amount of F_R_ and F_IR_ has been represented in Figure 12b. It has been found that the M2 membrane exhibited a higher 30.9% reversible fouling (F_R_) than the M1 membrane with a 22.2% F_R_ value. However, the percentage of irreversible fouling (F_IR_) for the M2 membrane is 14.7%, which is less compared to the M1 membrane showed an F_IR_ value of 28.3%, which is twice the M2. 

The obtained values of the F_T_, F_RR_, F_R_ and F_IR_ conclude that compare to the TFC M1 membrane, the TFN M2 membrane surface exhibits less amount of total fouling (F_T_) and a higher fraction of it is reversible, also the TFN M2 membrane has a better antifouling tendency as well. The smaller value of F_IR_ for the TFN M2 membrane represents the tendency of its surface to stick weakly with fouling materials [52,53].

### 4.4. Antibacterial Activity of Prepared TFC and TFN Membranes

**Figure 13:**
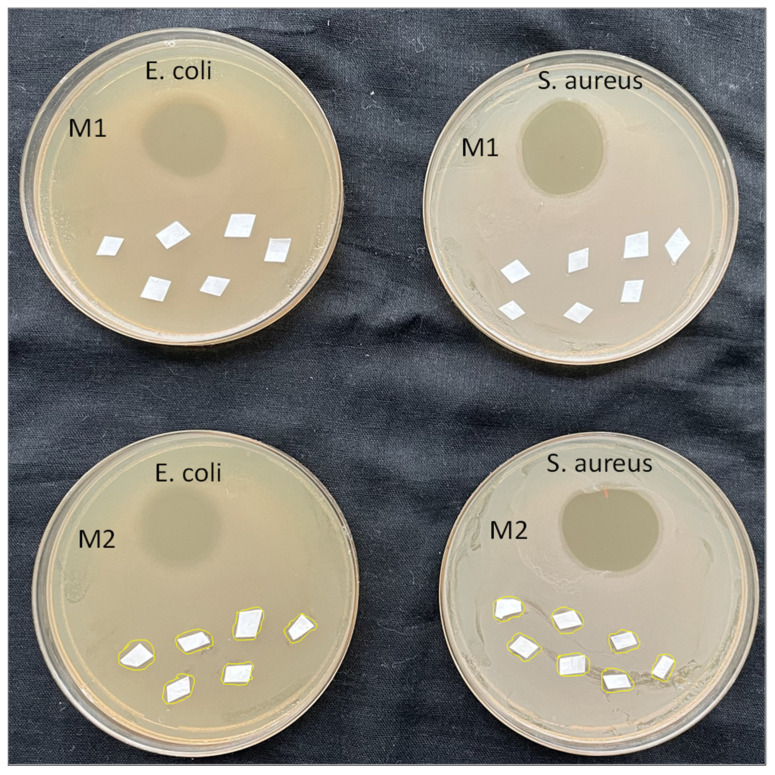
The antibacterial results of the M1 and M2 membrane after being subjected to 24 h with *E. coli* and *S. aureus*.

The antibacterial study of the prepared TFC and TFN membranes was carried out by the bacterial colony growth method against Gram-negative *E. coli.* (*Escherichia coli*) and Gram-positive *S. aureus* (*Staphylococcus aureus*) bacteria to check whether the membrane has antibacterial activity or not. From our previous work, the involvement of mesoporous synthetic hectorite (MSH) mixed UiO-66-NH_2_ nanoparticles has shown good antibacterial activity. In the current experiment, Figure 13 displays the antibacterial results of prepared membranes after subjecting with bacteria for 24 h. The aminoglycoside antibiotic named “Neomycin” was also used as a positive control to show activity against bacteria and used as the reference standard. The antibiotic exhibited a superior circular inhibition zone which is visible in Figure 13. The white sheets are the pieces of the membranes spread over the agar. A close look at the images of the M1 and M2 membrane in Figure 13 depicts that there is some retardation while colony-forming which is visible around the M2 membrane. Whereas, the M1 membrane has no mitigation against both the bacteria. The M2 membranes have shown mild antibacterial activity. It can be expected that the M2 membrane has interrupted the cells of the bacteria and due to the metal oxides present on the nanomaterials, the oxidative stress has further enhanced the antibacterial activity. The M2 membrane’s results have shown that the incorporated nanomaterial has developed antibacterial properties on the membrane [54]. The M2 membrane retarded *S. aureus* in its surrounding area, which is marked with yellow lines, showing the membrane’s inhibition zone. However, weak retardation of *E. coli.* was observed by the M2 membrane. The nanomaterial was loaded in a very small amount in the TFN M2 membrane, so it can be expected that the higher loading of nanomaterial may lead to better antibacterial activity.

## 5. Conclusions

Thin-film composite (M1 membrane) and Thin-film nanocomposite (M2 membrane) membranes come under the category of nanofiltration which is the pressure-driven membrane that does effective separation of dyes and ions by size exclusion and the Donnan ion phenomenon. In this experiment, we tested our prepared TFC and TFN membranes against very low concentrated malachite green dye and phosphate ions at higher pressure. The high pressure and low concentrated feed solution is the situation where membranes are triggered to their limit. From testing, we have obtained good results of dye separation from only a 100 ppm solution. The phosphate removal was measured from a very less concentrated 10 ppm solution which is harder to remove, and we obtained ~78% rejection with 22.22 Lm^−2^h^−1^ permeability from TFN membrane (M2 membrane). Compared to the dye separation the difference between the M1 and M2 membrane’s separation ability is greater while rejecting phosphate ions. The reasons can be the different phenomena that were more prominent during the separations; size exclusion while dye removal and Donnan effect during phosphate removal. The M1 (TFC) membrane has already been exhibiting good separation of dye which also does not leave a big room for enhancement in rejection with nanomaterial addition. 

The permeability of the membrane varies with the type of feed because the feed solutions properties such as density, viscosity, dielectric point, polarity etc. create a difference and as a result, we observed different permeability with different feed solutions. A solution with low viscosity will have greater permeation. The more hydrophilic surface also represents higher permeation as cleared from the contact angle values and the obtained permeation data. A more hydrophilic surface also provides a good antifouling tendency as seen in the antifouling experiment where the M2 membrane showed an enhanced 83.8% F_RR_ and 14.7% F_IR_ value. The antibacterial testing revealed that the nanomaterial addition caused the antibacterial activity in thin film nanocomposite (TFN) M2 membrane and which can further be increased if we increase nanomaterials concentration.

## Figures and Tables

**Table 1:** Datasheet of the composition of prepared TFC (thin-film composite) and TFN (thin-film nanocomposite) membranes.

S. No.	Membrane Name	PIP Conc. (wt %)	NPs Conc. (wt %) UiO-66-NH_2_	TMC Conc. (wt %)	Reaction Time (min)
1.	M1	2.0	-	0.2	1
2.	M2	2.0	0.02	0.2	1

**Table 2:** Contact angle measurements of polysulfone and prepared M1, M2 membrane.

Membrane	Contact Angle (°)	Images
PSf	89.1° (±2°)	** 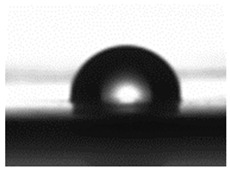 **
M1	69.5° (±2°)	** 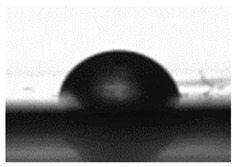 **
M2	58.4° (±2°)	** 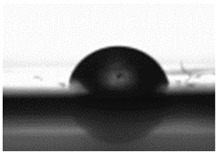 **

## Data Availability

Data will be made available on request.
